# Left Atrial Appendage Aneurysm Presenting With Atrial Fibrillation in a Young Woman: A Case Report

**DOI:** 10.1002/ccr3.73941

**Published:** 2026-11-14

**Authors:** Rezayat Parvizi, Shahla Meshgi, Mohammad Rostamzadeh, Sohrab Negargar, Eissa Bilehjani, Solmaz Fakhari, Amir Faravan

**Affiliations:** ^1^ Cardiovascular Research Center Tabriz University of Medical Sciences Tabriz Iran; ^2^ Department of Anesthesiology, Faculty of Medicine Tabriz University of Medical Sciences Tabriz Iran; ^3^ Student Research Committee Tabriz University of Medical Sciences Tabriz Iran

**Keywords:** aneurysm, atrial fibrillation, cardiac surgical procedures, left atrial appendage

## Abstract

Left atrial appendage aneurysm (LAAA) is a rare cardiac anomaly. We report a 25‐year‐old woman with LAAA presenting with atrial fibrillation and dyspnea. Multimodality imaging enabled diagnosis, and successful surgical resection was performed. Histopathology confirmed a benign aneurysm. At 6‐month follow‐up, no recurrence or thromboembolic events were observed.

## Key Clinical Message

Left atrial appendage aneurysm is a rare but treatable cause of atrial fibrillation and cardiomegaly in young adults. Multimodality imaging enables accurate diagnosis and surgical planning, while early resection may be considered to limit disease progression and reduce the risk of thromboembolic complications.

## 1 Introduction

Left atrial appendage aneurysm (LAAA) represents one of the rarest congenital cardiac anomalies, yet it carries life‐threatening potential due to its association with atrial arrhythmias, dyspnea, and thromboembolic events [1]. The anomaly is fundamentally attributed to abnormal development or a congenital weakness of the pectinate muscles within the appendage wall [2]. While surgical resection is generally accepted as the standard of care and pharmacologic therapy largely focuses on the prevention and control of thromboembolism and atrial tachyarrhythmias [3], alternative strategies, including percutaneous closure and catheter ablation, have also been reported as feasible treatment options [1]. Nevertheless, the extreme rarity of this condition introduces considerable diagnostic and therapeutic challenges, and the optimal management approach still requires careful deliberation supported by the documentation of new clinical experiences. The present case report describes a young woman with an LAAA that manifested as atrial fibrillation and was successfully treated with surgical resection.

## 2 Case History/Examination

A 25‐year‐old woman was referred to a cardiac surgery center with recurrent palpitations and exertional dyspnea of several months' duration. She had no prior history of congenital heart disease, rheumatic heart disease, stroke, cardiac surgery, or other significant underlying medical conditions. Physical examination revealed a heart rate of approximately 120 beats per min, with otherwise unremarkable findings. Electrocardiography demonstrated atrial fibrillation with a rapid ventricular response (Figure 1). Chest radiography revealed cardiomegaly and prominence of the left cardiac border (Figure 2). The overall clinical course is summarized in Table 1.

**FIGURE 1:**
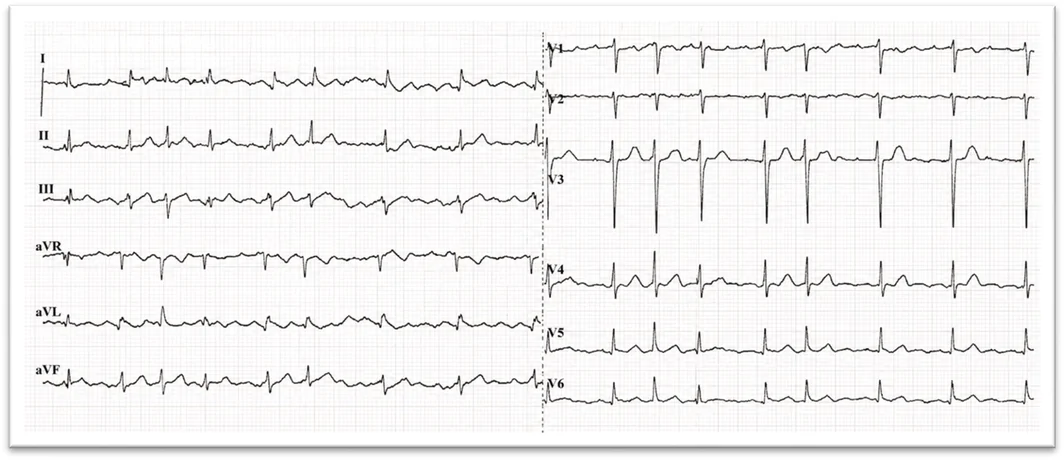
Preoperative 12‐lead electrocardiogram demonstrating atrial fibrillation rhythm.

**FIGURE 2:**
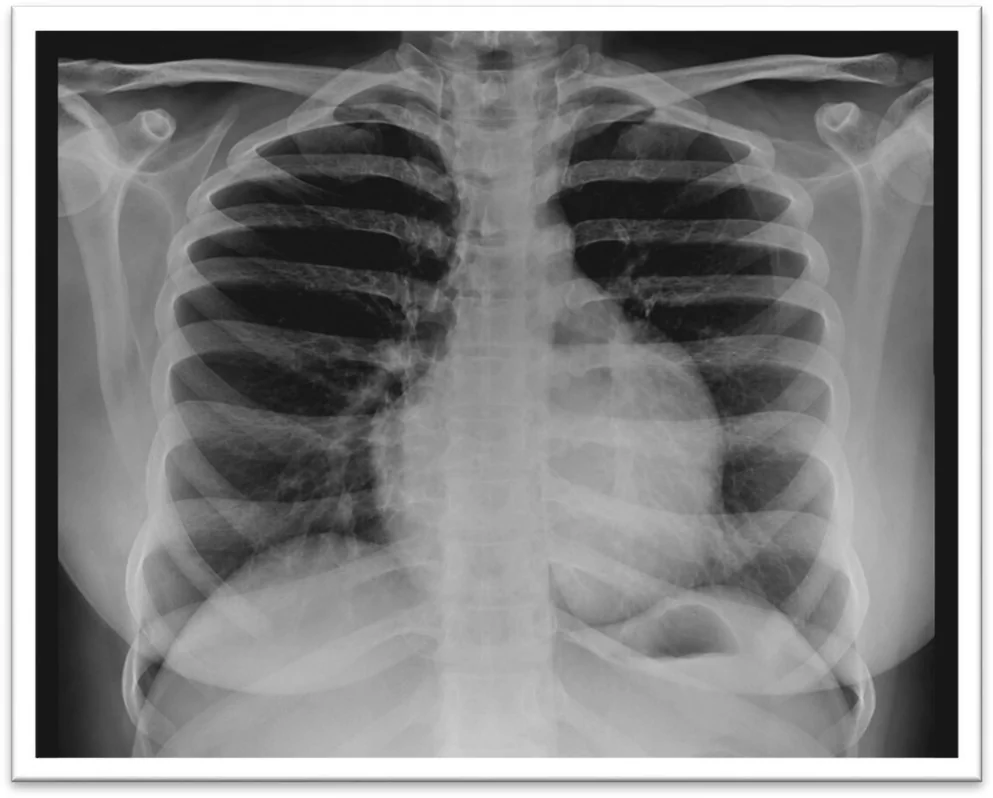
Chest radiograph shows cardiomegaly, prominence of the left cardiac border, and widening of the subcarinal angle.

**TABLE 1:** Timeline of the clinical course.

Time point	Key event
Several months before referral	Palpitations and exertional dyspnea.
Presentation	AF with rapid ventricular response; cardiomegaly on chest radiography.
Initial evaluation	TTE: LAAA 63 × 31 mm; LVEF 50%; no intracavitary thrombus or associated septal defect.
Further imaging	Cardiac CT: LAAA 80 × 67 × 62 mm; 24.7‐mm neck; normal pulmonary venous drainage.
Surgery	Complete surgical resection of LAAA via sternotomy; no concomitant Maze or surgical pulmonary‐vein isolation.
Discharge	Uneventful recovery; discharged on warfarin for persistent AF.
6‐month follow‐up	No thromboembolism, recurrence, or readmission; AF persisted.
Within first postoperative year	Catheter ablation performed; sinus rhythm restored.

Abbreviations: AF, atrial fibrillation; LAAA, left atrial appendage aneurysm; LVEF, left ventricular ejection fraction; TTE, transthoracic echocardiography.

## 3 Differential Diagnosis, Investigations, and Treatment

Transthoracic echocardiography was performed while the patient was tachycardic, with a heart rate of 122 bpm. Left ventricular dimensions were within the normal range (LVEDD 4.8 cm and LVESD 3.4 cm). Left ventricular systolic function was mildly reduced, with an LVEF of 50%, although this value may have been influenced by the rapid heart rate. The left atrial diameter was 3.6 cm, and the left atrial volume was 89 mL. The right atrium appeared normal in size. The inferior vena cava was also normal in size and showed more than 50% inspiratory collapse. Mild mitral and tricuspid regurgitation were present, with a tricuspid regurgitation gradient of 20 mmHg. Pulmonary artery pressure was estimated at 25 mmHg and was within the normal range. No atrial septal defect, ventricular septal defect, or anomalous pulmonary venous drainage was identified. The main echocardiographic finding was a left atrial appendage aneurysm measuring approximately 63 × 31 mm, without evidence of intracavitary thrombus.

Contrast‐enhanced cardiac CT angiography demonstrated mild cardiomegaly and an LAAA, confined to the left atrial appendage rather than involving the left atrium itself, arising from the inferior wall of the left atrium and extending superiorly. The aneurysm measured 80 × 67 × 62 mm and communicated with the left atrium through a 24.7‐mm neck (Figure 3). The larger dimensions observed on CT compared with echocardiography likely reflect the superior spatial resolution and three‐dimensional assessment provided by CT angiography. The left pulmonary veins were displaced posteriorly by the aneurysm, with otherwise normal pulmonary venous drainage (Figure 4).

**FIGURE 3:**
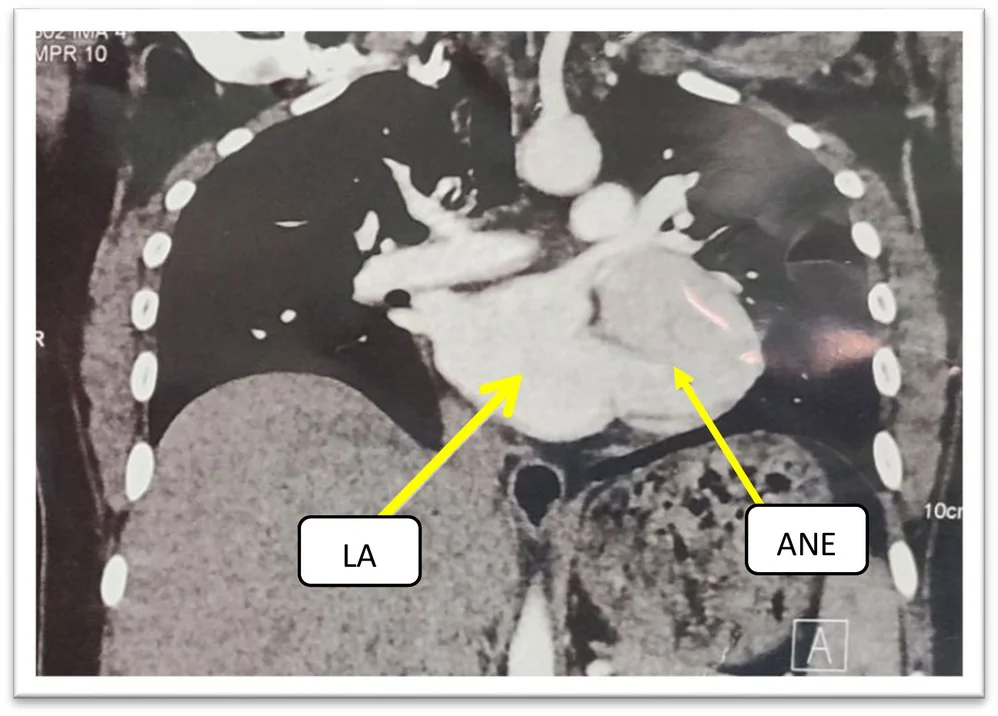
Cardiac CT angiography, coronal view, demonstrating a left atrial appendage aneurysm originating from the left atrium and measuring 80 × 67 × 62 mm. ANE, aneurysm; LA, left atrium.

**FIGURE 4:**
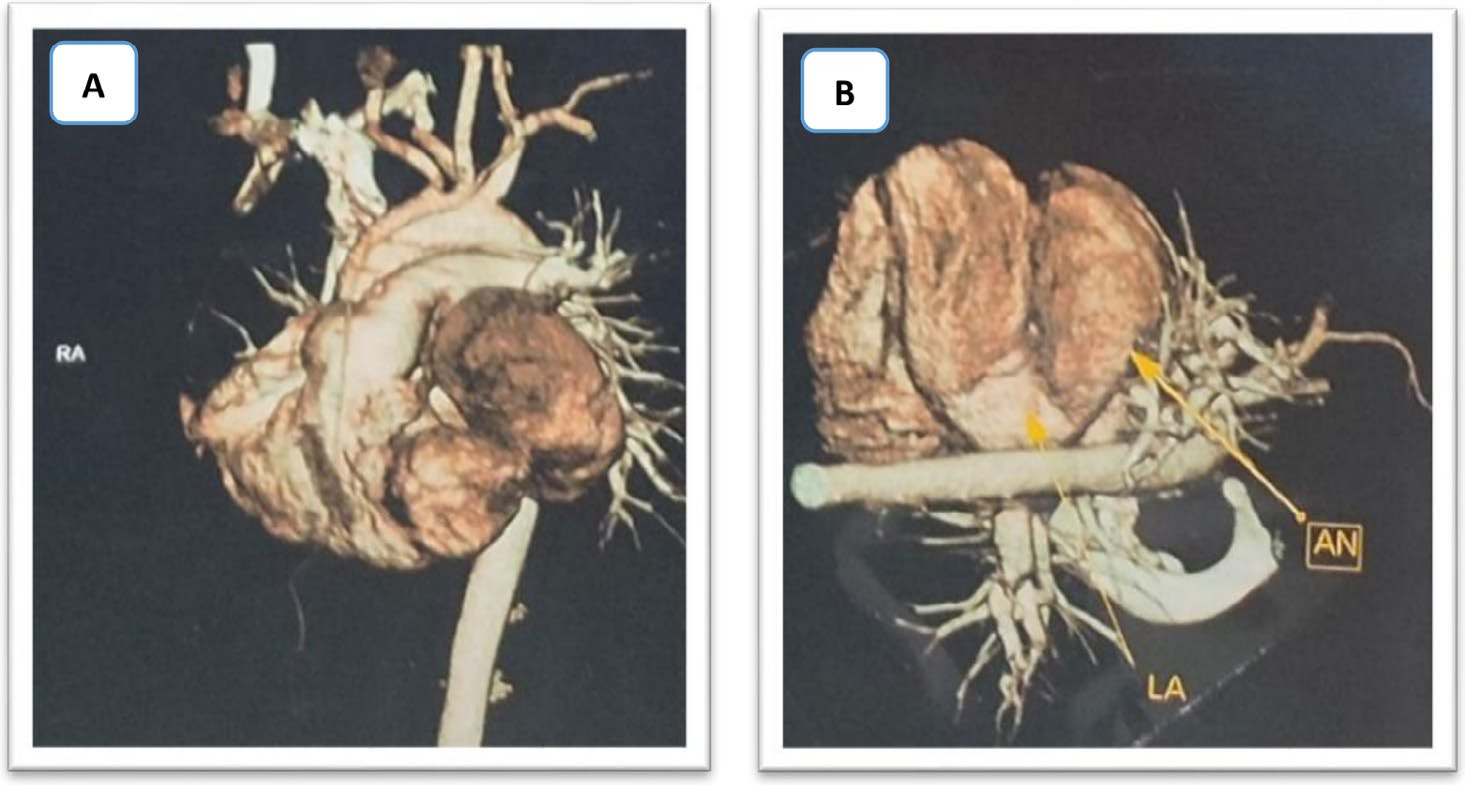
Three‐dimensional volume‐rendered cardiac CT angiography showing a left atrial appendage aneurysm. (A) Anterior view. (B) Oblique view.

Given the large size of the aneurysm and the presence of atrial fibrillation, surgical intervention was undertaken. The patient underwent surgery under general anesthesia through a sternotomy approach. After opening the pericardium, an LAAA was identified. The neck of the aneurysm was carefully dissected and completely mobilized. Following secure double‐layer closure of the aneurysmal neck, the aneurysmal sac was excised in its entirety. Gross examination of the resected specimen revealed a large, thin‐walled aneurysmal sac (Figure 5). The operation was completed successfully without intraoperative complications.

**FIGURE 5:**
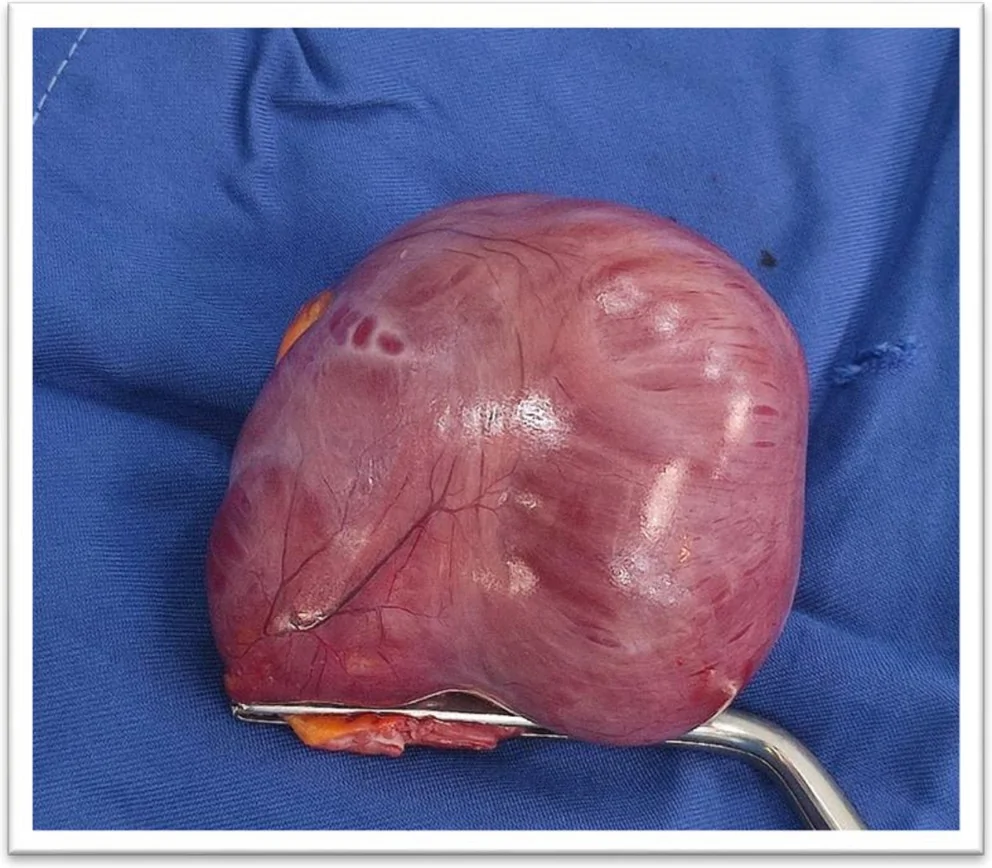
Gross specimen of the excised LAAA, demonstrating a thin‐walled aneurysmal sac following complete surgical resection.

## 4 Outcome and Follow‐Up

Histopathological analysis of the excised specimen revealed a thin‐walled fibromuscular aneurysmal sac with no evidence of malignancy, vasculitis, organized thrombus, or other specific pathological changes, confirming the diagnosis of congenital LAAA. The postoperative course was uneventful, and the patient was discharged in stable condition on warfarin therapy because of persistent atrial fibrillation. No concomitant surgical rhythm‐control procedure, including a Maze procedure or surgical pulmonary‐vein isolation, was performed at the time of aneurysm resection. Follow‐up transthoracic echocardiography demonstrated complete exclusion of the aneurysm, with no residual communication between the left atrium and the resection site, no pericardial effusion, and stable biventricular function without significant interval change. At 6‐month follow‐up, the patient remained clinically stable without thromboembolic events, aneurysm recurrence, or hospital readmission; however, atrial fibrillation persisted. During the first postoperative year, the patient subsequently underwent catheter ablation, after which sinus rhythm was successfully restored.

## 5 Discussion

LAAA is an extremely rare cardiac anomaly with potential life‐threatening consequences, primarily due to its association with atrial arrhythmias, thromboembolic events, and progressive cardiac remodeling. In large aggregated analyses, this condition has been reported predominantly in young adults with a slight female preponderance and a mean age in the third decade of life [1, 3]. The clinical presentation is often non‐specific, with palpitations and dyspnea being the most frequent symptoms, while atrial tachyarrhythmias such as atrial fibrillation or flutter commonly serve as the first major diagnostic clue [3, 4]. The present case is consistent with these findings, as the patient was a young woman presenting with palpitations and atrial fibrillation.

From a pathophysiological standpoint, LAAA is thought to arise from congenital weakness or abnormal development of the pectinate muscles of the left atrial appendage wall, although acquired forms have also been described, with the latter being more prevalent [2, 5]. Structural remodeling of the aneurysmal wall may contribute to electrical heterogeneity and conduction discontinuity, thereby predisposing patients to atrial fibrillation, flutter, or focal atrial tachycardia [6, 7, 8]. This mechanism likely explains the persistent atrial fibrillation observed in our patient even after surgical intervention.

Imaging plays a central role in diagnosis. Transthoracic and transesophageal echocardiography remain the most widely used first‐line modalities, while cardiac CT and MRI provide superior anatomical definition and thrombus assessment [3, 9]. In large case series, echocardiography was used in the majority of patients, with CT and MRI serving as adjunct tools for detailed structural evaluation and surgical planning. In the present case, multimodality imaging clearly demonstrated an aneurysm with a narrow neck and significant displacement of adjacent cardiac structures, consistent with previously reported morphologic patterns [10].

The mean reported size of LAAA in pooled analyses is approximately 7 cm, with some variation depending on imaging modality and population studied [1, 3]. Our case exceeded this average, representing an aneurysm with substantial hemodynamic and structural impact. Such large lesions are more likely to be associated with atrial arrhythmias and thromboembolic complications, particularly in the presence of atrial fibrillation [3].

Thromboembolic events remain one of the most serious complications of LAAA. Older age and atrial arrhythmias have been identified as significant predictors of thrombus formation and systemic embolization [1]. In untreated cases, systemic embolism and atrial tachyarrhythmias represent the most frequently reported complications, and atrial fibrillation or flutter is considered the strongest predictor of embolic risk [3]. Although our patient did not present with thromboembolic complications, surgical management was undertaken based on the overall clinical presentation and aneurysm characteristics.

Management strategies for LAAA vary depending on symptoms, aneurysm size, and presence of complications. While conservative management may be considered in asymptomatic patients without thrombus, surgical resection remains the treatment of choice in most reported cases due to its excellent outcomes and definitive resolution of embolic risk [1, 11]. Innovative approaches including minimally invasive resection, catheter ablation, and device‐based closure (e.g., WATCHMAN FLX) have also been reported with favorable short‐term outcomes in selected patients [9]. In the present case, surgical excision via sternotomy was successfully performed without complications, consistent with the high success rates reported in the literature.

Interestingly, although surgical treatment is highly effective in preventing embolic events, restoration of sinus rhythm is not guaranteed in all patients. Persistent atrial fibrillation after resection, as observed in our case, has also been described in previous reports and may reflect underlying atrial remodeling prior to intervention [12]. This observation provides a rationale for considering timely treatment as a potential means of limiting further atrial remodeling, although restoration of sinus rhythm cannot be expected from aneurysm resection alone.

LAAA may also be associated with other congenital anomalies such as atrial septal defects, ventricular septal defects, anomalous renal arteries, and Noonan syndrome, emphasizing the need for comprehensive evaluation of associated structural abnormalities [3]. Furthermore, incidental detection on echocardiography or CT imaging is common, reinforcing the importance of careful interpretation of cardiac imaging studies [10].

Overall, this case is consistent with previous reports showing that LAAA is a rare but clinically important condition associated with atrial arrhythmias and thromboembolic complications. Multimodality imaging is important for accurate diagnosis and surgical planning. Timely intervention may help reduce the risk of disease progression and embolic complications, although persistence of atrial fibrillation after resection remains possible.

## 6 Conclusion

Left atrial appendage aneurysm should be considered in young patients presenting with unexplained cardiomegaly and atrial fibrillation. Multimodality imaging is essential for diagnosis and surgical planning, and early surgical treatment may be considered to reduce the risk of progressive atrial remodeling and thromboembolic complications.

## Author Contributions

**Rezayat Parvizi:** investigation, data curation, writing – original draft. **Shahla Meshgi:** investigation, visualization, writing – review and editing. **Mohammad Rostamzadeh:** investigation, data curation. **Sohrab Negargar:** validation. **Eissa Bilehjani:** supervision, resources. **Solmaz Fakhari:** validation, writing – review and editing. **Amir Faravan:** conceptualization, project administration, supervision, writing – original draft, writing – review and editing.

## Funding

The authors have nothing to report.

## Ethics Statement

The study was conducted in accordance with institutional and national ethical standards and the Declaration of Helsinki.

## Consent

Written informed consent was obtained from the patient for the publication of the clinical information and accompanying images. All identifying patient information has been completely anonymized to protect the patient's privacy.

## Conflicts of Interest

The authors declare no conflicts of interest.

## Data Availability

The data that support the findings of this study are available from the corresponding author upon reasonable request.
